# Coping mechanisms adopted following a suicide attempt among refugees in humanitarian settings within northern Uganda

**DOI:** 10.1017/gmh.2026.10263

**Published:** 2026-06-25

**Authors:** Moses Mukasa, Roscoe Kasujja, Benjamin Alipanga, Gwendolyn Portzky, Wouter Vanderplasschen

**Affiliations:** 1 Special Needs, Ghent University, Belgium; 2 Mental Health and Community Psychology, Makerere University, Uganda; 3 Makerere University, Uganda; 4 Department of Mental Health and Community Psychology, Makerere University, Uganda; 5 Makerere University College of Humanities and Social Sciences, Uganda; 6 Ghent University, Belgium

**Keywords:** coping mechanisms, suicide attempt, Suicide survivors Northern Uganda, Refugees, Refugees of South Sudanese origin

## Abstract

Most suicides happen following maladaptive coping among suicide survivors; however, coping mechanisms adopted by refugee suicide survivors, especially in Uganda, have hardly been studied. This study assessed the coping mechanisms adopted following suicide attempts among refugees in humanitarian settings in Northern Uganda. A concurrent mixed-methods design was used to study adult refugee-suicide survivors of South Sudanese origin. They were consecutively sampled across four settlements and engaged in structured, in-depth interviews. Data were analyzed in SPSS version 25 using descriptive statistics, while qualitative data were analyzed thematically. Fewer than a quarter (17%) of suicide attempts were coped with adaptively. Most refugees coped emotionally by rarely accepting sympathy and understanding (37.5%), frequently trying to keep their feelings to themselves (50.0%), self-blame (50.0%), self-isolation (62.5%), making a plan to act (37.5%), bursting out in anger and other emotions (75.0%), and having fantasies or wishes about how things might turn out (62.5%). These coping mechanisms were congruent with those identified in the qualitative exploration. Refugees in Northern Uganda with a history of suicide attempts maladaptively cope with that history, implying that they could be at high risk of repeated suicide attempts and potentially suicide, based on evidence shown in previous studies.

## Graphical abstract

**Figure:**
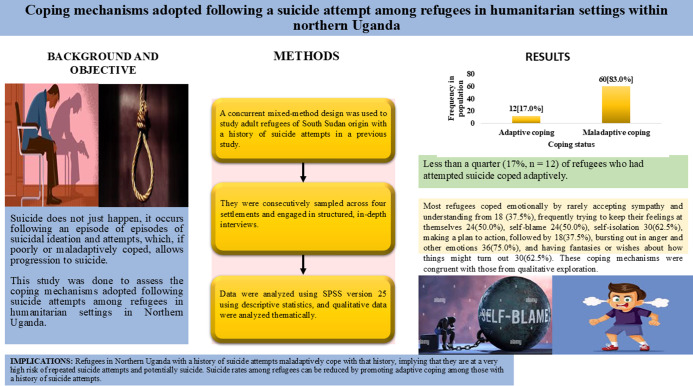

## Impact statement

Sub-Saharan Africa also hosts the largest population of refugees, at 5.4 million, renowned for being among the most vulnerable to suicide, and concurrently comprises 48 of the 108 low- and middle-income countries that registered the highest suicide rates in 2021. This is evidence of the need to implement more suicide prevention efforts across Africa, one of which is the promotion of adaptive coping for all refugees with a history of suicidal ideation or attempts. Suicide follows episodes of suicidal ideation and attempts, which, if poorly or maladaptively coped with, allow progression to suicide. Thus, coping mechanisms, which are behaviors or thoughts adopted to manage stressful conditions that can be external or internal, are significant for suicide prevention. However, the assessment of coping mechanisms, while extensively conducted before, has not included refugees, especially those with a history of suicide attempts. This is also true in Uganda, which hosts Africa’s largest refugee population, with a substantial suicide rate, which might proliferate even more if the transition from a suicide attempt to suicide is not prevented through education on how to cope adaptively with the consequences of a suicide attempt. We believe that this study is among the first to assess coping mechanisms adopted following a suicide attempt in a refugee context, and it could be significant for mental health and psychosocial support programs, particularly if published in the Cambridge Prisms journal and is widely accessed. Consequently, the findings might indicate a new angle that can be taken by MHPSS programs in Uganda and other host countries in their efforts to curb suicide in humanitarian settings. Even better, international organizations such as UNHCR, the Danish Refugee Council, and the Jesuit Refugee Service might also base their MHPSS programming models on the findings, including a component for promoting adaptive coping.

## Introduction

A suicide occurs every minute (World Health Organization [WHO], 2025), making it a significant public health challenge that warrants more attention than it currently receives. Moreover, the global suicide rate has not decreased significantly over the past 20 years; during that period (between 2000 and 2021), the rate decreased by 35%, translating to approximately 1.7% per year. The smallest reduction occurred between 2022 and 2023 (Ritchie, 2026), when the rate remained almost stationary. A mean suicide reduction rate of 1.7% per year will not be sufficient to achieve indicator 3.4.2 (33% reduction in the suicide rate by 2030 compared with 2015) of target 3.4 (reduction of premature mortality from non-communicable diseases) (WHO, 2026). Previous evidence indicates that the age-standardized suicide rate decreased from 9.0 per 100,000 people in 2019 to only 8.9 per 100,000 people in 2021 (International Association for Suicide Prevention [IASP], 2022), a difference of only 0.1%. Regionally, most suicide deaths occur in Eastern Europe (19.2 deaths per 100,000), followed by Southern Sub-Saharan Africa (16.1 per 100,000), and Central Sub-Saharan Africa (14.4 per 100,000) (Weaver et al., 2025). Sub-Saharan Africa reported the highest suicide mortality in 2021 and hence significantly contributes to the global suicide burden. The fact that Sub-Saharan Africa comprises 48 of the 108 (44%) countries in the low- and middle-income country categorization also implies that it significantly contributes to the reported 547,500 annual cases of suicide registered in those countries. Given the social stigma and cultural concerns that lead to concealment, the number of suicide cases may be higher than previously reported (Dattani et al., 2023). Another alarming fact is that sub-Saharan Africa is home to the world’s second-largest population, estimated at 1.54 billion people (World Population Review, 2026). It is also host to the largest population of refugees, at 5.4 million, renowned for being among the most vulnerable to suicide (Cogo et al., 2022; Bevione et al., 2024). This is further evidence for the implementation of more suicide prevention efforts in Africa, which can take several routes. Among the most important yet usually sidelined of those is the promotion of adaptive coping for all refugees with a history of suicidal ideation or attempts. This is premised on evidence that suicide follows episodes of suicidal ideation and attempts (Weissman et al., 2026), which, if poorly or maladaptively coped with (using emotion-based coping), can allow progression to suicide. Coping mechanisms refer to thoughts, perceptions, emotions and behaviors that are adopted by a given individual to reduce the effects or manage internal or external stressful conditions or situations (Folkman and Moskowitz, 2004; Ruvalcaba et al., 2023; Algorani and Gupta, 2023). Some authors (Cooper et al., 2006) have categorized coping mechanisms into problem-focused (adaptive) and emotion-focused (maladaptive) mechanisms. Problem-focused coping involves active adoption of activities that aim at directly tackling the problem/stressor. Emotion-focused coping, on the other hand, involves the use of emotional support, religion, avoidance, and humor to try to reduce the effects of the stressor, without necessarily removing the stressor (Baloran, 2020; Nurunnabi et al., 2020; Savitsky et al., 2020; Garcini et al., 2022). In essence, emotional coping following a suicide attempt does not enable one to address the explanatory cause of the previous suicide attempt, but rather lets it remain prominent, to the extent of causing further suicidal ideation. That is why it is called maladaptive coping, since it does not allow for growth of adaptive capacity to deal with the previous stressor and prevent its progressive effect of suicidal ideation and inclination toward another potentially successful suicide attempt (Garg et al., 2022; Okechukwu et al., 2022). This denotes the significance of promoting the adoption of adaptive coping mechanisms among all refugees with a suicide attempt history, especially in countries like Uganda, which hosts Africa’s largest refugee population. Besides hosting such a large refugee population, the country has perennially registered high suicide rates, especially in Northern Uganda (Athumani, 2021; UNHCR, 2023; UNHCR, 2025). However, the promotion of coping mechanisms requires that evidence is generated indicating the status of coping among refugees with suicide attempt history. But, despite the extensiveness of studies on refugees and their suicidal behaviors, even in Uganda (e.g., Kizza et al., 2012; Athumani, 2021; Bukuluki et al., 2021), there were barely any that assessed coping mechanisms adopted following a suicide attempt. This study was conducted to assess the coping mechanisms adopted following suicidal ideation and suicide attempts among refugees in humanitarian settings in Northern Uganda.

## Methodology

### Overview of study methodology

**Figure 1.**
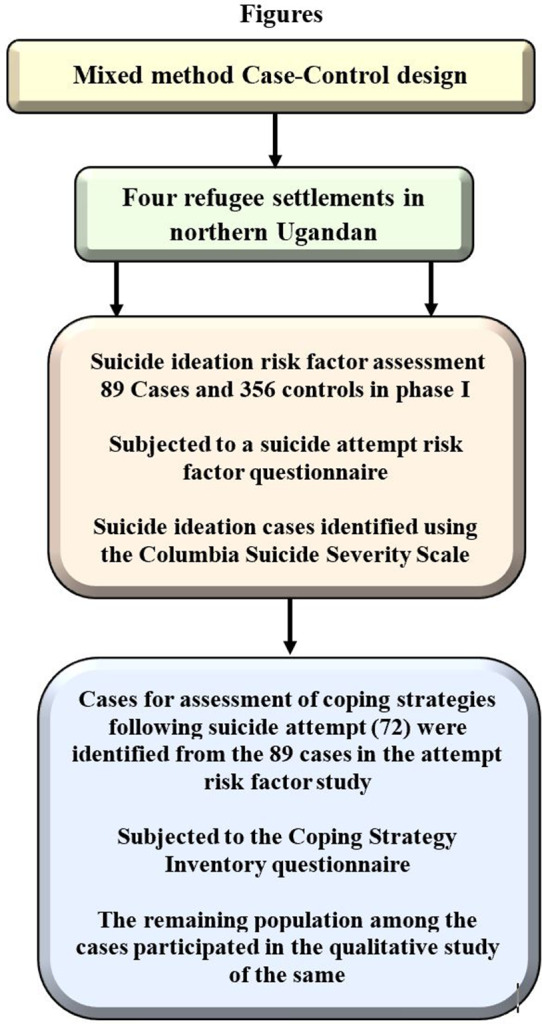
Overview of study methodological approach. Figure 1. long description.

This study is a sequel to a mixed-method case–control study that was conducted to assess the risk factors and coping mechanisms adopted following suicidal ideation and attempts among refugees in four refugee settlements in the northwest Nile, Uganda (Rhino camp, BidiBidi, Palorinya, and Nyumanzi refugee settlements). Initially, activities to collect data for the assessment of suicide attempt risk factors were mounted. Each of the four settlements was stratified by zone, allowing for a simple random sampling of villages and systematic random sampling of households within each village (Figure 1). Quantitatively, 89 cases and 356 controls of suicide attempts were identified and studied for the risk factor assessment with the help of the Columbia Suicide Severity Scale, which has already demonstrated high reliability in screening for suicidal behaviors (Posner et al., 2011; Nam et al., 2024). Thus, in the current study on coping following a suicide attempt, 89 suicide attempt cases were the population size used to compute the number of refugees required. This calculation, performed using the Krejcie and Morgan formula, yielded 72 suicide attempts that were subjected to the Coping Strategy Inventory questionnaire for quantitative coping strategy assessment (Figure 1). Some of the remaining cases that did not participate in the quantitative assessment of the coping strategy for suicidal ideation were purposively sampled to participate in the qualitative segment of the study.

### Study design and area

This study adopted a concurrent mixed-method design, characterized by the collection of both quantitative and qualitative data, albeit not in a sequence. The quantitative component of this study involved the assessment of coping mechanisms among 72 refugees with a history of suicide attempts. The qualitative component involved exploring the coping mechanisms adopted by the 17 cases not included in the quantitative assessment. The qualitative data were collected using a phenomenological approach, since coping mechanisms are lived experiences of persons with a history of attempted suicide (Giorgi, 1985; Boss et al., 1996; Bassett, 2004). This design was used to conduct a study across four refugee settlements in Northern Uganda, where South Sudanese refugees settled. They included Imvepi, Parolinya, Bidi Bidi, and Rhino Camp refugee settlements, all of which registered more than 70% of suicide attempt cases that were reported between January 2023 and August 2023 in Uganda (UNHCR, 2023). These settlements were chosen as study areas because while refugees in Uganda have been reported to register a high prevalence of suicide, most of those cases are contextual to refugee settlements in Northern Uganda (UNHCR, 2020; Athumani, 2021; UNHCR, 2023).

### Study participants

The study population consisted of adult *refugees of South Sudanese origin who had a history of suicide attempt, as earlier identified in the suicide attempt risk factor study. Those refugees participated in both the quantitative and qualitative arms of the study, wherein they responded to an exploration of coping mechanisms for suicide attempts. The study included adult refugees who declared (verbatim) willingness to have a suicide history assessed during the consent process. Only such refugees were included in this study because those who were free to divulge accurate information about their attempt history ensured the study’s high credibility and reliability. Qualitative study participants who were uncomfortable with their responses being digitally recorded were excluded because voice recording is the only way to validate and comprehensively capture qualitative study responses (Lee, 2004; Al-Yateem, 2012; Nordstrom, 2015).

### Sample size determination

For the quantitative study, the required sample size was determined using the formula by Krejcie and Morgan (1970), as the population size (*N*) of suicide ideation cases had already been established. The formula is given by *s* = X^2^N P (1 − P)/d^2^ (N − 1) + X^2^ P (1 − P), which was true when *s* = required sample size; *X* ^2^ = the table value of chi-square for one degree of freedom at the desired confidence level (3.841), *N* is the population size = number of refugees who had suicidal ideations, as identified in the ideation risk factor study = 96; *P* = the population proportion (assumed to be 0.50, as this would provide the maximum sample size); and *d* is the degree of accuracy expressed as a proportion (0.05). This yielded a sample size of 72 refugees with a history of suicide attempts. However, in a qualitative study, data saturation was used to determine the number of respondents who were to be included in the qualitative exploration of coping mechanisms, as per Guest (2006). In-depth interviews were conducted until data saturation was reached, as indicated by response overlap (Morse, 1995; Guest et al., 2006; Morse, 2015). With this approach, saturation occurred at the 14th in-depth interview.

### Sampling procedures

Given that this assessment required 72 participants for the quantitative assessment of coping mechanisms adopted following a suicide attempt, across the four settlements, consecutive sampling was used to sample the respondents. Consecutive sampling was used because the sample size of refugees with a history of suicide attempt required in this study was about 80% of the population size earlier predetermined, implying that the use of random sampling was impractical. Random sampling, despite being renowned for reducing sampling bias, involves probabilistic elimination and requires larger population sizes to be used. Consecutive sampling is the least biased of all the qualitative sampling methods (Polit and Beck, 2017) and does not involve probability sampling, meaning that it could allow for the obtainment of the required sample size, with respect to the small population size available. Consecutive sampling involves the inclusion of any respondent for as long as they meet the inclusion criteria of the study (Polit and Beck, 2017). The sampling was applied following the establishment of the number of refugees that was required from each settlement, by proportioning the sample size (72) according to the population size of the cases in each settlement.

For the qualitative study, whose available population size was predetermined as being 17, purposive sampling was used to sample three participants per refugee settlement. This strategy was used to ensure that at the point of data saturation, at least all four refugee settlements would be represented. It happened that data saturation was not reached until the time when 12 in-depth interviews had been held across the four settlements (first round of interviews). Therefore, another round was conducted, in which data saturation was reached when three interviews were conducted in Rhino camp, making a total of 15 interviews that had been conducted by the time data saturation set in.

### Data collection techniques

Structured interviews were conducted to collect data for the quantitative assessment of coping mechanisms. These interviews were ideal for the quantitative study because the adapted Coping Strategies Inventory (CSI) was structured, requiring administration in a standardized manner. Qualitative data, on the other hand, were collected using in-depth interviews, which are conducted one-on-one and are well suited to exploring sensitive issues, such as coping mechanisms following suicidal ideation. The principal investigator moderated all in-depth interviews to increase the likelihood of accurately detecting data saturation. Responses from structured interviews were captured using a structured questionnaire comprising items from the CSI, which contains 71 items (Speyer et al., 2016). The CSI identifies emotion-focused engagement, emotion-focused disengagement, problem-focused engagement, and problem-focused disengagement. The CSI has demonstrated acceptable internal consistency (α = 0.56–0.80) in countries such as the United Kingdom (UK), the United States (US), Australia, Sweden, and Germany, making it suitable for assessing coping strategies. The CSI tool was translated into Juba-Arabic and Dinka, two of the most widely spoken and understood languages among South Sudanese refugees in Uganda. Qualitative data generated from in-depth interviews were captured using an in-depth interview guide and were also audio-recorded.

### Measurement of coping strategies and suicide attempt

The participants in this study had earlier been screened for suicide attempt history in the attempt risk factor study, wherein Columbia Severity Rating Scale (C-SRS) was used. The C-SSRS has already demonstrated high reliability in screening for suicidal behaviors (Posner et al., 2011; Nam et al., 2024). Posner et al. (2011) reported that the C-SSRS tool showed very high divergent and convergent validity with other suicidal behavior screening scales, along with a very high level of specificity and sensitivity. Nam et al. (2024) recently concluded that the Cronbach’s alpha of the C-SSRS ranged from 0.74 to 0.89, proving its reliability.

Coping strategies were measured using 72 indicators as prescribed in the CSI tool, each of which had attributes that represented adaptive or maladaptive coping inclinations. Adaptive coping represented problem-focused coping practice adoption, while maladaptive coping represented more emotion-focused coping practice adoption. The responses per question were Not at all, A Little, Somewhat, Much, and Very much, which were scored 1, 2, 3, 4, and 5, respectively. However, that scoring was for problem-focused assertions; for emotion-focused assertions, the scoring was reversed, as Not at all = 5, A Little = 4, Somewhat = 3, Much = 2, Very much = 1. The reverse scoring was premised on the fact that emotion-focused coping is discouraged as it breeds further depression and increases progression to suicide volition. For problem-focused assertions, the responses which showed satisfactory coping frequency were “much and very much,” while for emotion-focused coping, the responses which showed satisfactory frequency of adopting a given coping activity were “Not at all” and “A Little.” In essence, the minimum score for a refugee to be deemed as having adaptively coped was 288 (4 × 72), with the maximum score being 360 (5 × 72), implying that the refugee had to score between 288 and 360 to be considered as being an adaptive coper of suicide attempt history.

### Statistical and qualitative data analysis

Quantitative data management and analyses were performed using SPSS version 25. Descriptive analysis was performed using frequency distributions, followed by principal component and cluster analyses. Principal component and cluster analyses were performed to identify the most important components defining coping mechanisms, given that the questionnaires used to assess both variables had numerous items that needed to be dimensionally reduced. To determine whether the data on coping mechanisms, as obtained from the assessment with the CSI, met the assumptions for analysis using principal component analysis (PCA), the Kaiser–Meyer–Olkin (KMO) measure and Bartlett’s test of sphericity were run first. This test, as shown below, turned out to be statistically significant (*p* < 0.05), with the KMO measure of Sampling Adequacy being above the acceptable limit of 0.6, at 0.673. This confirmed that the data were suitable for dimensionality reduction, and it was hence subjected to PCA.

**Table 1.** Table 1. long description.

KMO Measure of Sampling Adequacy.		0.673
Bartlett’s Test of Sphericity	Approx. Chi-Square	45.119
	Df	3
	Sig.	0.000

PCA was performed using the varimax method with orthogonal rotation (Jolliffe, 2016). All variables with eigenvalues exceeding one were considered principal components. However, to ascertain the distribution of the 11 principal components that were identified from PCA, cluster sampling was performed using K-means cluster analysis (Lloyd, 1957; MacQueen, 1967), with which the number of clusters required was set at two (2), with 10 maximum iterations. The distance between cases and cluster centers was set using the simple Euclidean distance. This resulted in the establishment of the largest cluster of refugees with a given set of coping mechanisms (previously identified as principal components). Each principal component was then cross-tabulated with the cluster to determine the distribution of the principal components in the largest cluster and the coping mechanisms adopted by the majority of respondents who had ideated or attempted suicide. These characteristics in the largest cluster defined the coping mechanisms among the studied suicidal ideation cases.

Qualitative data generated from the exploration of coping mechanisms were analyzed using Braun and Clarke’s (2006) thematic analysis approach. The analysis was conducted through a six-phase process, which was preceded by transcription of recorded responses to text by the principal investigator. This was then followed by familiarization with the data in Phase 1, generation of initial codes in Phase 2, search for themes in Phase 3, review of themes in Phase 4, and definition and naming of themes in Phase 5. For each theme generated, the principal investigator conducted a detailed analysis of the quotes therein, identifying the story each theme told (Braun and Clarke, 2006) and adding a narrative. This was done to show what each theme was about and how it was interlinked with other themes and the study.

## Results

### Sociodemographic characteristics of respondents in the quantitative assessment of coping mechanisms adopted following a suicide attempt

Table 2 shows that three-quarters 54 (75.0%) of the refugees were female and were aged between 40 and 50 years (54, 75.0%). Almost all refugees were married (66, 91.7%), although the majority had not received any formal education (54, 75.0%). Among those who had received formal education, 18 (100.0%) had completed primary education. Most of the refugees were Christian (Anglican), 54 (75.0%); not currently employed, 66 (91.7%); and had stayed in their respective settlements for 5 to 10 years, 54 (75.0%).

**Table 2.** Sociodemographic characteristics of participants in the assessment of coping strategies following suicide attempt Table 2. long description.

Variable	*n*	%
**Gender**		
Female	54	75.0
Male	18	25.0
**Current age in years**		
Between 29 and 39	6	8.3
Between 40 and 50	54	75.0
More than 50 years	12	16.7
**Current marital status**		
Married	66	91.7
Widow	6	8.3
**Received any formal education**		
Yes	18	25.0
No	54	75.0
**Level of formal education received**		
Primary	18	100.0
**Religious denomination**		
Christian (Catholic)	12	16.7
Christian (Pentecost)	6	8.3
Christian (Anglican)	54	75.0
**Employed currently**		
Yes	6	8.3
No	66	91.7
**Nationality**		
South Sudanese	72	100.0
**Duration of stay in the settlement**		
Between 5 and 10 years	54	75.0
More than 10 years	18	25.0

### Classification of coping mechanisms in the assessment of coping mechanisms adopted following a suicide attempt

**Figure 2.**
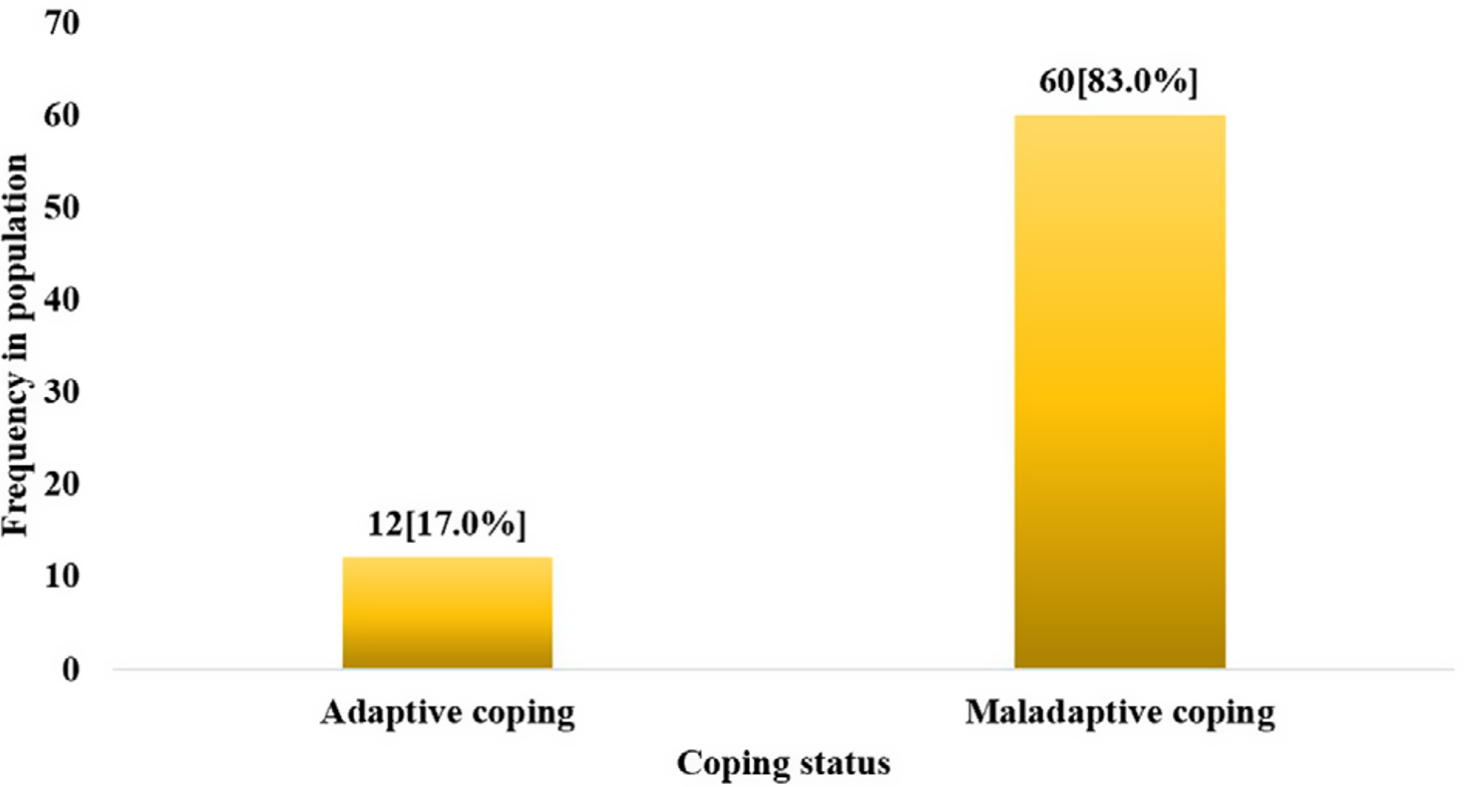
Distribution of coping status among suicide survivors.

Figure 2 shows that the assessment of coping mechanisms using the coping strategy inventory revealed that less than a quarter (17%, *n* = 12) of refugees who had attempted suicide coped adaptively.

### Identification of the most important coping mechanisms (PCA)

PCA was performed on the 71 coping strategies reported by the refugees to determine which of those were most correlated and, hence, could indicate a definitive picture of how refugees cope after a suicide attempt. This process resulted in the identification of 11 principal components (Table 3), that is, those with eigenvalues of 1 and above. These components were letting their emotions out (coef. 0.957), wishing that they had not let themselves become involved in the situation (coef. 0.841), accepting sympathy and understanding from others (coef. −0.882), trying to maintain feelings of the self (coef. 0.911), making a plan of action, and following it (coef. 0.879), indicating that one had brought problems to oneself (coef. 0.824), and spending more time alone (coef. 0.942), being angry or really blowing up (coef. 0.889), standing ground, and fighting what one wants (coef. 0.869), criticizing oneself for what has happened (coef. 0.698), and having fantasies or wishes about how things might turn out (coef. 0.410).

**Table 3.** Rotated matrix from the PCA of coping strategies adapted following a suicide attempt Table 3. long description.

Coping strategy	Principal component number										
	1	2	3	4	5	6	7	8	9	10	11
I just concentrated on what I had to do next; the next step	0.009	−0.596	−0.088	−0.335	−0.038	0.603	0.004	−0.268	0.284	0.031	−0.079
I tried to get a new angle on the situation	0.651	0.298	0.030	0.011	0.083	−0.423	−0.393	0.112	−0.062	0.338	0.125
I found ways to blow off steam	0.378	−0.310	0.118	0.134	0.616	0.244	0.351	−0.223	0.342	0.000	−0.005
I accepted sympathy and understanding from someone	0.208	−0.104	**−0.882**	−0.101	−0.117	−0.074	−0.062	−0.289	−0.047	−0.083	0.204
I slept more than usual	0.714	−0.112	0.196	0.063	0.319	0.093	0.017	−0.201	0.361	0.360	−0.157
I hoped the problem would take care of itself	−0.312	0.295	0.532	0.463	0.260	−0.077	0.027	−0.414	0.115	0.218	0.107
I told myself that if I were not careless, things like this would not happen	0.716	−0.469	0.212	0.162	0.233	0.135	−0.241	0.250	0.002	−0.045	−0.009
I tried to keep my feelings to myself	−0.135	0.245	0.056	**0.911**	0.186	−0.110	−0.050	0.147	−0.092	0.084	0.039
I changed something so that things would turn out all right	0.920	−0.017	−0.148	0.032	−0.126	0.282	−0.008	−0.135	0.008	−0.133	−0.005
I looked for the silver lining, so to speak, and tried to look on the bright side of things	0.888	0.166	0.109	0.089	0.151	0.058	−0.084	0.144	0.109	0.287	0.126
I did some things to get it out of my system	0.709	−0.190	0.377	−0.004	0.435	0.034	0.177	0.168	0.203	0.143	0.087
I found somebody who was a good listener	0.604	0.164	−0.113	0.182	0.635	−0.106	−0.164	−0.079	0.192	0.271	0.071
I went along as if nothing were happening	0.278	−0.082	0.245	0.832	0.150	−0.320	0.054	−0.125	−0.093	−0.040	0.101
I hoped a miracle would happen	−0.923	0.030	0.182	0.084	−0.080	−0.004	0.120	−0.098	0.121	0.247	−0.013
I realized that I brought the problem on myself	0.399	0.008	0.044	0.201	0.150	**0.824**	−0.178	0.236	0.078	−0.050	−0.034
I spent more time alone	0.043	−0.257	0.076	−0.039	0.119	0.102	**0.942**	−0.009	−0.086	−0.051	−0.049
I stood my ground and fought for what I wanted	0.286	−0.136	0.002	−0.125	0.254	0.024	0.057	−0.244	**0.869**	−0.005	0.035
I told myself things that helped me feel better	0.695	0.397	−0.174	−0.128	0.240	−0.106	−0.030	0.358	−0.148	0.304	0.016
I let my emotions go	0.467	−0.163	−0.024	−0.102	0.494	0.100	−0.634	0.220	−0.019	0.112	0.164
I talked to someone about how I was feeling	0.812	−0.020	−0.011	−0.424	−0.076	0.023	−0.016	0.119	0.183	0.326	0.010
I tried to forget the whole thing	0.704	0.435	−0.224	−0.235	−0.082	−0.068	−0.243	−0.053	0.072	0.355	0.069
I wished that I had not let myself get involved with that situation	−0.006	**0.841**	0.291	0.120	−0.189	−0.006	−0.003	−0.311	−0.133	0.022	0.208
I blamed myself	−0.161	0.294	−0.245	0.802	−0.283	0.261	−0.123	0.051	−0.106	0.086	0.000
I avoided my family and friends	−0.408	0.772	−0.336	−0.051	0.169	−0.095	0.190	0.193	−0.007	0.045	−0.097
I made a plan of action and followed it	−0.055	−0.002	0.198	0.072	**0.879**	0.086	0.033	0.352	0.150	−0.051	−0.151
I looked at things in a different light and tried to make the best of what was available	0.766	0.172	0.296	0.144	0.354	−0.153	−0.094	−0.055	−0.178	0.248	−0.147
I let out my feelings to reduce the stress	0.755	0.265	0.445	−0.108	0.047	0.015	0.277	−0.005	−0.187	−0.025	0.190
I spent more time with people I liked	0.884	−0.215	−0.123	−0.115	0.087	−0.119	−0.032	−0.109	0.298	0.136	0.036
I did not let it get to me; I refused to think about it too much	0.640	0.302	0.499	−0.089	−0.027	−0.129	0.155	−0.341	0.112	0.203	0.176
I wished that the situation would go away or somehow be over with	0.164	0.695	0.045	0.391	0.406	0.238	−0.081	−0.168	0.110	0.212	0.148
I criticized myself for what happened	0.232	0.188	−0.038	0.488	−0.030	0.146	−0.395	0.061	−0.055	**0.698**	−0.014
I avoided being with people	−0.262	0.696	−0.178	0.003	−0.118	0.292	0.522	−0.037	−0.046	0.107	0.171
I tackled the problem head-on	0.599	0.327	−0.077	−0.051	0.361	−0.076	0.006	0.460	0.405	−0.046	0.108
I asked myself what was really important, and discovered that things were not so bad after all	0.533	−0.023	−0.130	−0.191	0.180	−0.014	0.021	−0.384	0.383	0.552	0.175
I let my feelings out somehow	0.542	0.111	0.317	−0.180	0.085	0.355	0.135	−0.012	**0.601**	0.065	−0.210
I talked to someone who I was very close to	0.696	0.019	−0.372	−0.149	−0.205	0.330	−0.164	0.193	0.299	0.141	−0.176
I decided that it was really someone else’s problem and not mine	0.131	0.023	0.509	0.506	0.479	0.245	0.067	0.355	0.010	−0.157	−0.153
I wished that the situation had not started at all	−0.362	0.201	0.025	−0.221	0.243	−0.295	−0.379	0.667	−0.159	0.139	−0.014
Since what happened was my fault, I really chewed myself out	−0.061	0.844	0.223	0.342	−0.033	0.069	−0.232	−0.075	−0.174	−0.061	−0.135
I did not talk to other people about the problem	−0.611	0.156	0.038	0.652	0.082	0.084	−0.181	−0.314	0.006	0.035	−0.173
I knew what had to be done, so I doubled my efforts and tried harder to make things work	0.290	0.558	0.262	−0.272	−0.168	−0.053	0.096	−0.582	0.283	0.054	−0.020
I convinced myself that things are not quite as bad as they seem	0.924	0.068	−0.078	0.094	0.052	0.010	−0.063	−0.015	0.240	−0.233	−0.092
I let my emotions out	**0.957**	−0.013	0.113	−0.074	0.032	0.101	−0.182	0.059	0.059	0.116	−0.015
I let my friends help out	0.943	−0.119	−0.266	0.079	−0.056	−0.013	−0.092	0.011	0.020	0.072	0.054
I avoided the person who was causing trouble	0.726	−0.051	0.032	0.548	−0.090	0.250	0.085	0.289	−0.024	−0.037	−0.068
I had fantasies or wishes about how things might turn out	0.734	0.222	0.297	−0.021	−0.104	0.168	0.266	−0.083	0.086	0.177	**0.410**
I realized that I was personally responsible for my difficulties and really lectured myself	0.388	−0.163	0.477	0.350	−0.431	−0.208	−0.041	0.247	−0.217	0.107	0.351
I spent some time by myself	−0.051	0.226	0.235	−0.079	0.154	−0.159	0.879	0.174	0.152	−0.016	0.095
It was a tricky problem, so I had to work around the edges to make things come out OK	0.357	0.176	0.338	−0.388	0.360	0.464	−0.417	0.230	0.010	−0.062	−0.042
I stepped back from the situation and put things into perspective	0.094	0.706	0.029	0.086	0.192	0.092	−0.251	0.439	0.244	−0.353	0.006
My feelings were overwhelming, and they just exploded	0.361	0.209	−0.304	0.320	0.399	0.578	0.176	−0.094	0.044	0.241	0.195
I asked a friend or relative I respect for advice	0.789	−0.012	−0.332	0.031	−0.238	0.276	−0.177	0.119	0.052	0.062	−0.285
I made light of the situation and refused to get too serious about it	0.910	0.001	0.137	−0.115	−0.095	0.189	0.076	−0.032	−0.053	−0.282	−0.084
I hoped that if I waited long enough, things would turn out OK	0.572	−0.213	0.374	0.422	−0.324	−0.205	0.191	0.172	−0.186	−0.226	−0.103
I kicked myself for letting this happen	−0.086	−0.731	0.346	0.454	0.318	−0.025	−0.024	−0.120	0.047	−0.118	0.026
I kept my thoughts and feelings to myself	−0.274	−0.002	0.330	−0.357	0.180	0.468	0.598	−0.004	0.215	−0.119	0.138
I worked on solving the problems in the situation	0.427	0.335	0.307	−0.602	−0.193	−0.210	−0.059	0.031	−0.146	0.039	0.374
I reorganized the way I looked at the situation, so things did not look so bad	0.854	−0.216	0.272	−0.095	−0.183	−0.094	0.084	−0.012	0.274	0.065	0.110
I got in touch with my feelings and just let them go	0.755	0.215	0.282	−0.237	−0.251	0.288	0.273	0.005	0.086	0.063	0.129
I spent some time with my friends	0.733	−0.350	0.192	0.254	0.000	0.177	−0.269	0.284	−0.126	−0.068	0.184
Every time I thought about it, I got upset, so I just stopped thinking about it	0.511	0.170	−0.624	0.091	0.247	0.427	−0.049	−0.043	−0.087	0.227	−0.071
I wished I could have changed what happened	0.049	0.384	−0.054	−0.067	−0.387	0.676	0.110	0.076	−0.242	0.395	−0.059
It was my mistake, and I needed to suffer the consequences	−0.374	0.404	0.205	0.022	0.386	0.580	0.386	0.065	−0.064	−0.100	0.025
I did not let my family and friends know what was going on	−0.465	−0.121	−0.076	0.082	0.851	0.005	0.154	0.021	−0.024	0.014	0.084
I struggled to resolve the problem	0.383	0.752	0.037	0.241	0.024	0.054	−0.101	−0.093	0.144	0.407	−0.140
I went over the problem again and again in my mind and finally saw things in a different light	0.270	0.613	−0.094	−0.493	−0.135	−0.129	0.412	0.231	−0.096	−0.179	0.013
I was angry and really blew up	0.379	−0.049	−0.069	0.151	−0.008	0.061	0.135	**0.889**	−0.111	−0.034	−0.037
I talked to someone who was in a similar situation	0.263	−0.424	0.806	−0.212	−0.132	0.099	−0.025	−0.149	0.025	−0.060	0.041
I avoided thinking or doing anything about the situation	0.440	−0.451	0.290	−0.188	0.195	0.617	0.099	0.033	0.169	−0.122	0.106
I thought about fantastic or unreal things that made me feel better	0.594	−0.045	0.440	−0.377	−0.061	0.172	0.342	−0.002	0.083	0.026	0.389
I told myself how stupid I was	0.480	−0.220	0.348	−0.024	0.451	0.367	−0.053	0.037	0.263	−0.334	−0.277
I did not let others know how I was feeling	0.045	0.205	0.806	0.247	0.279	0.057	0.332	−0.010	0.000	−0.116	0.200

The bold values indicate the highest coeficient in each of the principal component columns, indicating the adjacent variable that was identified as the textual descriptor of the given component.

### Determination of the largest cluster to which the respondents belonged

**Figure 3.**
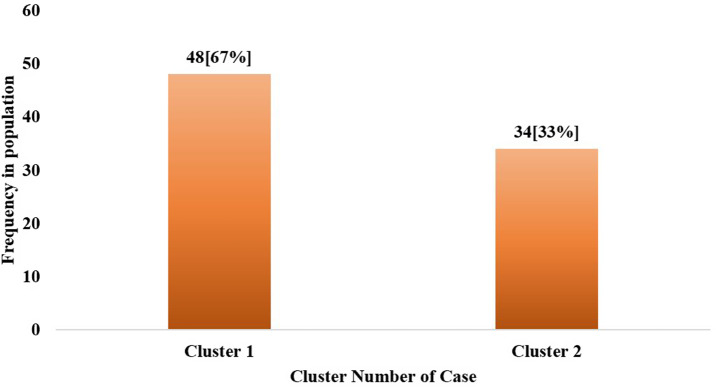
Cluster membership of the 10 principal components.

Using the *K*-means clustering approach, cluster analysis (Figure 3) revealed that refugees who participated in the quantitative assessment of coping mechanisms adopted after a suicide attempt belonged to two clusters. The largest of these clusters was Cluster 1, comprising 48 (67%) of them (Figure 3). Therefore, this study aimed to determine the coping mechanisms adopted by refugees in the largest cluster. It is the coping mechanism in the largest cluster that was taken as representative of most refugees and, hence, the most definitive.

### Determination of coping mechanisms in the largest cluster of refugees

To determine the coping mechanisms adopted by most refugees, a cross-tabulation of each of the 11 principal components identified with the two clusters was conducted (Table 4). The findings in Table 3 show that in the largest cluster of refugees who had attempted suicide, the largest proportion coped by rarely accepting sympathy and understanding from (18 [37.5%]), frequently trying to keep their feelings to themselves (24 [50.0%]), realizing that they brought the problem to themselves (18 [37.5%]), spending more time alone (30 [62.5%]), standing on their ground and fighting for what they wanted (24 [50.0%]), wishing that they had not allowed themselves to get involved with the situation (24 [50.0%]), and making a plan to action followed by (18 [37.5%]). Many of them were angry and really blowing up (36 [75.0%]), criticizing themselves for what happened 24 (50.0%), letting their emotions out (30 [62.5%]), and having fantasies or wishes about how things might turn out (30 [62.5%]).

**Table 4.** Disaggregation of the 11 principal components into the largest cluster Table 4. long description.

Component	Cluster number	
	1 (One)	2 (Two)
**I accepted sympathy and understanding from someone**		
Not at all	**18 (37.5%)**	0 (0.0%)
A Little	6 (12.5%)	12 (50.0%)
Somewhat	18 (37.5%)	0 (0.0%)
Much	6 (12.5%)	12 (50.0%)
**I tried to keep my feelings to myself**		
Somewhat	6 (12.5%)	0 (0.0%)
Much	18 (37.5%)	18 (75.0%)
Very much	**24 (50.0%)**	6 (25.0%)
**I realized that I brought the problem on myself**		
Somewhat	12 (25.0%)	0 (0.0%)
Much	**18 (37.5%)**	24 (100.0%)
Very much	**18 (37.5%)**	0 (0.0%)
**I spent more time alone**		
A Little	6 (12.5%)	6 (25.0%)
Somewhat	0 (0.0%)	12 (50.0%)
Much	**30 (62.5%)**	6 (25.0%)
Very much	12 (25.0%)	0 (0.0%)
**I stood my ground and fought for what I wanted**		
Not at all	0 (0.0%)	6 (25.0%)
A Little	0 (0.0%)	6 (25.0%)
Somewhat	**24 (50.0%)**	0 (0.0%)
Much	18 (37.5%)	12 (50.0%)
Very much	6 (12.5%)	0 (0.0%)
**I wish that I had not at all let myself get involved with that situation**		
A Little	0 (0.0%)	6 (25.0%)
Somewhat	18 (37.5%)	0 (0.0%)
Much	**24 (50.0%)**	6 (25.0%)
Very much	6 (12.5%)	12 (50.0%)
**I made a plan of action and followed it**		
Not at all	12 (25.0%)	0 (0.0%)
A Little	12 (25.0%)	0 (0.0%)
Somewhat	6 (12.5%)	12 (50.0%)
Much	**18 (37.5%)**	12 (50.0%)
**I was angry and really blew up**		
Somewhat	**36 (75.0%)**	12 (50.0%)
Much	12 (25.0%)	6 (25.0%)
Very much	0 (0.0%)	6 (25.0%)
**I criticized myself for what happened**		
A Little	12 (25.0%)	0 (0.0%)
Somewhat	12 (25.0%)	6 (25.0%)
Much	**24 (50.0%)**	6 (25.0%)
Very much	0 (0.0%)	12 (50.0%)
**I let my emotions out**		
A Little	6 (12.5%)	0 (0.0%)
Somewhat	6 (12.5%)	12 (50.0%)
Much	**30 (62.5%)**	12 (50.0%)
Very much	6 (12.5%)	0 (0.0%)
**I had fantasies or wishes about how things might turn out**		
A Little	6 (12.5%)	0 (0.0%)
Somewhat	6 (12.5%)	18 (75.0%)
Much	**30 (62.5%)**	6 (25.0%)
Very much	6 (12.5%)	0 (0.0%)

The bold values indicate the highest coeficient in each of the principal component columns, indicating the adjacent variable that was identified as the textual descriptor of the given component.

### Qualitative findings from the assessment of coping mechanisms adopted following a suicide attempt

**Figure 4.**
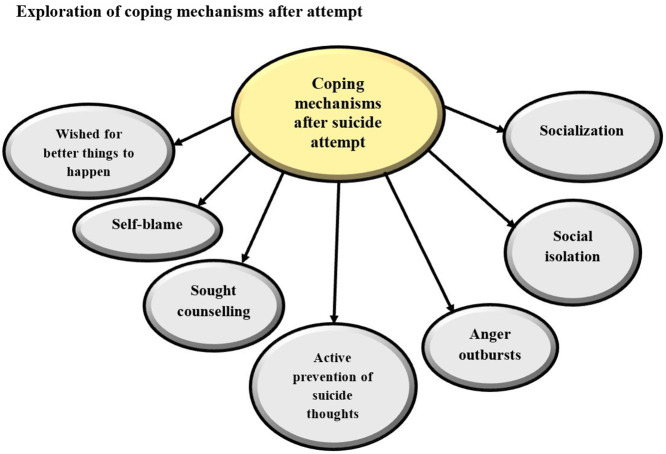
Thematic tree showing emergent themes from the analysis of coping mechanism adopted after a suicide attempt.

Figure 4 shows the findings from the exploration of coping mechanisms adopted following a suicide attempt, revealing four key themes: self-isolation, self-blame, active prevention of suicidal thoughts, and anger outbursts. Nonetheless, a few participants mentioned that they had adopted more coping mechanisms following their suicide attempts, as discussed below.

### Self-isolation

Many refugees tried to cope with their suicide attempts by isolating themselves from others. Most of them noted that they realized that being around people had made them internalize stigma and could exacerbate their ideations and inclinations to attempt suicide.

***………………I have kept to myself since; I do not like being around people because some of them got to know that I almost killed myself, and they stigmatize me, so I decided that I should not be around them, since it would make me get angry or more depressed. Male, In-depth interview 7, Parolinya.***

Some participants mentioned that staying away from other people in the community enabled them to heal, especially toxic people who had contributed to their suicidal nature.

***……………To a great extent, staying away from people is what has enabled me to get along since I attempted suicide. One of the things that made me attempt to take my life was being around toxic people, so I have decided to isolate myself whenever I can, for peace of mind. Female, in-depth interview 12, Rhino camp.***

### Self-blame

Self-blame was a rampantly mentioned coping mechanism that nearly all participants adopted. Most seemed to judge themselves for the suicide attempt they had made earlier, and decided to face any consequences henceforth.

*……………you see, I am the one who attempted suicide, so I have learnt to blame myself, and blame me, so that I feel guilty and try to heal, so that I do not attempt to commit suicide again. **Male, In-depth interview 8, Parolinya.***

### Active prevention of suicidal thoughts

Many participants also mentioned that they chose to prevent their suicidal thoughts actively. Some went ahead and mentioned the things they did to ensure that they practically or behaviorally tackled suicidal ideation and attempts. The practices they employed included cessation of alcohol consumption, seeking counseling, and many other factors that predisposed them to suicidality.

***………….For me, if it had not been for practically fighting suicidal thoughts and trying to engage the things that caused my suicidal ideation and attempt last time, I would have killed myself by now. I was an alcoholic last time, thinking that the alcohol would enable me to reduce the depression, but it got worse, so, to prevent any further bad things from happening, I have chosen to withdraw from alcohol consumption, and other things that I think might predispose me to being suicidal again. Male, In-depth interview 9, Nyumanzi refugee settlement.***

### Anger outbursts and venting

Similar to coping mechanisms for suicidal ideation, many refugees mentioned that they coped by letting out their anger and quarreling most of the time. One of the refugees with such an approach to coping mentioned that letting out their anger made them feel better, and ideated or planned to attempt suicide less.

**……………*I have coped by being free to do what I want, especially by getting angry and showing that I am actually angry, through letting my voice be heard and quarreling. It actually sounds weird, but letting out my feelings has made me cope with the fact that I attempted suicide; it could be that the last time, I was keeping all my emotions in, and it made me get more depressed.* Female, in-depth interview 6, BidiBidi refugee settlement.**

## Discussion

In summary, this study found that less than a quarter (17%) of the suicide attempts coped adaptively. Most refugees coped emotionally by rarely accepting sympathy and understanding from 18 (37.5%), frequently trying to keep their feelings at themselves 24 (50.0%), self-blame 24 (50.0%), self-isolation 30 (62.5%), making a plan to action, followed by 18 (37.5%), bursting out in anger and other emotions 36 (75.0%), and having fantasies or wishes about how things might turn out 30 (62.5%). These coping mechanisms were congruent with those identified in the qualitative exploration. For illustration, the 11 quantitatively identified coping mechanisms were categorizable into **Isolation** (frequently trying to keep their feelings to themselves, realizing that they brought the problem to themselves, and spending more time alone), **Actively preventing attempt causes** (standing on their ground and fighting for what they wanted), and **Self-blame** (wishing that they had not allowed themselves to get involved with the situation, and making a plan to action followed by, and having fantasies or wishes about how things might turn out). This was significantly congruent with the five qualitatively identified emergent coping mechanisms including self-isolation, self-blame, active prevention of suicidal thoughts, and anger outbursts. Therefore, this confirms the imperativeness of assessing coping mechanisms with a pragmatic research philosophy lens, as opposed to only qualitatively. That is because a pragmatic approach provides a bidirectional complementary value to quantitative and qualitative findings.

According to the transactional model of stress and coping, a person who faces a stressor, such as suicidal ideation and attempt, adopts coping mechanisms by default in an attempt to minimize the impact of that stressor. This was true among refugees who had ideations and attempted suicide, which is consistent with the findings of Al-Dajani et al. (2022), Liang et al. (2020), Stanley et al. (2021), Cao et al. (2020), Cai et al. (2020), and Muller et al. (2020), Parcesepe et al. (2023). Thus, as expected, refugees who had attempted suicide endeavored to minimize the consequences of their actions and, perhaps, avoid transition to graver outcomes such as suicide. However, worryingly, the majority of them maladaptively coped; the findings showed that less than a quarter (17%, *n* = 12) of refugees who had attempted suicide coped adaptively. This finding implies that only about two in every ten refugees who ideate or attempt suicide adopt problem-focused coping strategies or coping strategies that are proactive and involve focus on minimizing the effect of the stressor. In other words, most refugees in Northern Uganda adopt emotion-focused coping following a suicide attempt, characterized by stressor avoidance, which exacerbates the stressor and puts them at risk of transitioning to a suicide attempt. Although very few studies have assessed coping after suicide attempts among refugees, generic studies have confirmed a high prevalence of emotion-focused or maladaptive coping among persons with a history of suicide attempts (Konkan et al., 2014; Sun et al., 2021; Ge and Tolmie, 2025). Coping was performed by self-blaming, self-isolation, and bursting out in anger, which are typical of emotion-focused coping (Nurunnabi et al., 2020; Savitsky et al., 2020; Garcini et al., 2022) and are usually associated with subsequent substance abuse and impulsive behaviors (Jahan and Burgess, 2023; Otache et al., 2023). This is because, with emotion-focused coping, one simply sits back and presumes that the ideations will somehow subside on their own; they become more introverted, which gives room for intense loneliness, which only makes suicidal ideation and urges for suicide attempts more severe (Hernandez-Vasquez et al., 2019; Zhang et al., 2019; Bračič et al., 2019). Recent evidence also indicates that without good emotion regulation, suicidal ideation persists, increasing the risk of suicide attempts (Stetsiv et al., 2026), which can progress to suicide. One of the key outcomes or cardinal signs of emotion-focused coping is self-isolation, the outcome of which includes more intense distress (D’Amico et al., 2015; Fried et al., 2020; Lui et al., 2020), which are renowned precursors of psychological distress and suicidal ideation. Longstanding loneliness is associated with a greater likelihood of engaging in impulsive behaviors, such as substance use, as a secondary coping strategy (Rhew et al., 2021; Otache et al., 2023; Walsh and Schlauch, 2024). This further aggravates suicidal ideation severity, guaranteeing an effective transition from a suicide attempt. Only a few refugees were protected from these outcomes, and they included those who spent time with friends, though, on their own admission, they did so infrequently. The findings of the quantitative assessment of coping strategies largely concurred with those from the qualitative exploration. With the widespread maladaptive coping approaches used by suicidal refugees, it comes as no surprise that the number of suicide cases registered in Uganda coincided with the number of suicide attempt cases. The most recent analysis of suicide incidents among refugees and nationals in Uganda indicated that 27% of all suicide attempts by 2025 resulted in suicide (United Nations High Commissioner for Refugees, 2025). More concerning is the fact that there were nearly twice as many repeat suicide attempts (45) as first-time suicide attempts (25) among refugees (United Nations High Commissioner for Refugees, 2025), which is symbolic of maladaptive coping among refugees with a history of suicide attempts.

## Conclusion/implications

Most of the refugees (8 in every 10) with a history of attempted suicide maladaptively coped with the aftermath of their mental health challenges, implying that they are at a high risk of repeated suicide attempts and potentially suicide, given that they are not coping with the cause of the problem. The largest proportion of them cope by rarely accepting sympathy and understanding from someone frequently trying to keep their feelings to themselves, realizing that they brought the problem to themselves, spending more time alone, standing on their ground and fighting for what they wanted, wishing that they had not let themselves get involved with the situation, and making a plan to act and following it, being angry and really blowing up criticizing themselves for what happened, letting their emotions out, and having fantasies or wishes about how things might turn out. It is almost certain that without enabling more adaptive coping for all refugees with a history of suicide attempt, other psychosocial support interventions may be less effective. Thus, available mental health and psychosocial support interventions and those planned in the future, targeting refugees with an attempt history, ought to emphasize the need for education and support aligned toward enabling the adoption of more problem-focused coping. Such refugees should be dissuaded from blaming themselves for their predicament and self-isolation, and encouraged to address the root cause by seeking support and means to minimize triggers.

## Data Availability

The data on which the findings in this study are based are available upon reasonable request from the principal investigator.

## Long descriptions

### Figure 1. Long description

The flowchart consists of four sequential boxes connected by downward-pointing arrows.

1. The top box is yellow and labeled Mixed method Case-Control design.

2. An arrow points down to a light green box labeled Four refugee settlements in northern Ugandan.

3. Two arrows point down to a large beige box describing Phase I. It contains three points: Suicide ideation risk factor assessment 89 Cases and 356 controls in phase I; Subjected to a suicide attempt risk factor questionnaire; and Suicide ideation cases identified using the Columbia Suicide Severity Scale.

4. A final arrow points down to a large light blue box detailing the assessment of coping strategies. It contains three points: Cases for assessment of coping strategies following suicide attempt 72 were identified from the 89 cases in the attempt risk factor study; Subjected to the Coping Strategy Inventory questionnaire; and The remaining population among the cases participated in the qualitative study of the same.

Navigate back to Figure 1..

### Table 1. Long description

The table contains two main sections of statistical data. The first row shows the K M O Measure of Sampling Adequacy with a value of 0.673. The second section covers Bartlett’s Test of Sphericity, which includes three specific metrics: an Approx. Chi-Square value of 45.119, degrees of freedom (D f) of 3, and a significance (Sig.) value of 0.000.

Navigate back to Table 1..

### Table 2. Long description

The table consists of three columns: Variable, n, and percent.

* Gender: Female 54 (75.0 percent), Male 18 (25.0 percent).

* Current age in years: Between 29 and 39 is 6 (8.3 percent), Between 40 and 50 is 54 (75.0 percent), More than 50 years is 12 (16.7 percent).

* Current marital status: Married 66 (91.7 percent), Widow 6 (8.3 percent).

* Received any formal education: Yes 18 (25.0 percent), No 54 (75.0 percent).

* Level of formal education received: Primary 18 (100.0 percent).

* Religious denomination: Christian Catholic 12 (16.7 percent), Christian Pentecost 6 (8.3 percent), Christian Anglican 54 (75.0 percent).

* Employed currently: Yes 6 (8.3 percent), No 66 (91.7 percent).

* Nationality: South Sudanese 72 (100.0 percent).

* Duration of stay in the settlement: Between 5 and 10 years is 54 (75.0 percent), More than 10 years is 18 (25.0 percent).

Navigate back to Table 2..

### Table 3. Long description

The table presents a rotated matrix from a Principal Component Analysis P C A. The header row lists Principal component numbers from 1 to 11. The first column lists 66 specific coping strategy statements. Each row contains numerical factor loadings for that strategy across the 11 components.

Key high-loading values (bolded in the source) include:

* I accepted sympathy and understanding from someone: -0.882 in component 3.

* I tried to keep my feelings to myself: 0.911 in component 4.

* I realized that I brought the problem on myself: 0.824 in component 6.

* I spent more time alone: 0.942 in component 7.

* I stood my ground and fought for what I wanted: 0.869 in component 9.

* I wished that I had not let myself get involved with that situation: 0.841 in component 2.

* I made a plan of action and followed it: 0.879 in component 5.

* I criticized myself for what happened: 0.698 in component 10.

* I let my feelings out somehow: 0.601 in component 9.

* I was angry and really blew up: 0.889 in component 8.

* I let my emotions out: 0.957 in component 1.

* I had fantasies or wishes about how things might turn out: 0.410 in component 11.

Values range from approximately -0.923 to 0.957, representing the strength and direction of the relationship between each strategy and the underlying principal components.

Navigate back to Table 3..

### Table 4. Long description

The table consists of three columns: Component, Cluster number 1 (One), and Cluster number 2 (Two).

1. I accepted sympathy and understanding from someone: Cluster 1 peaks at Not at all and Somewhat (both 18, 37.5 percent). Cluster 2 peaks at A Little and Much (both 12, 50.0 percent).

2. I tried to keep my feelings to myself: Cluster 1 peaks at Very much (24, 50.0 percent). Cluster 2 peaks at Much (18, 75.0 percent).

3. I realized that I brought the problem on myself: Cluster 1 is split between Much and Very much (both 18, 37.5 percent). Cluster 2 is 100 percent at Much (24).

4. I spent more time alone: Cluster 1 peaks at Much (30, 62.5 percent). Cluster 2 peaks at Somewhat (12, 50.0 percent).

5. I stood my ground and fought for what I wanted: Cluster 1 peaks at Somewhat (24, 50.0 percent). Cluster 2 peaks at Much (12, 50.0 percent).

6. I wish that I had not at all let myself get involved with that situation: Cluster 1 peaks at Much (24, 50.0 percent). Cluster 2 peaks at Very much (12, 50.0 percent).

7. I made a plan of action and followed it: Cluster 1 peaks at Much (18, 37.5 percent). Cluster 2 is split between Somewhat and Much (both 12, 50.0 percent).

8. I was angry and really blew up: Cluster 1 peaks at Somewhat (36, 75.0 percent). Cluster 2 peaks at Somewhat (12, 50.0 percent).

9. I criticized myself for what happened: Cluster 1 peaks at Much (24, 50.0 percent). Cluster 2 peaks at Very much (12, 50.0 percent).

10. I let my emotions out: Cluster 1 peaks at Much (30, 62.5 percent). Cluster 2 is split between Somewhat and Much (both 12, 50.0 percent).

11. I had fantasies or wishes about how things might turn out: Cluster 1 peaks at Much (30, 62.5 percent). Cluster 2 peaks at Somewhat (18, 75.0 percent).

Navigate back to Table 4..
