# Supplementary material for: Coping mechanisms adopted following a suicide attempt among refugees in humanitarian settings within northern Uganda
Source: Glob Ment Health (Camb). 2026 Jun 25;13:e136. doi: 10.1017/gmh.2026.10263 (PMC13373283; doi:10.1017/gmh.2026.10263)
Supplement: Peer review history

# Reviewer report: Review: Coping mechanisms adopted following a suicide attempt among refugees in humanitarian settings within northern Uganda — R0/PR2

**DOI:** 10.1017/gmh.2026.10263.pr2

**Authors:** Frank Pio Kiyingi

**Affiliations:** Psychology and social sciences, Nkumba University, Uganda

The study is of current interest which addresses global and contextual issues in mental health and humanitarian settlings among refugees in Northern Unganda.

# Reviewer report: Review: Coping mechanisms adopted following a suicide attempt among refugees in humanitarian settings within northern Uganda — R0/PR3

**DOI:** 10.1017/gmh.2026.10263.pr3

The article addresses an important and clinically relevant topic and includes a reasonably large sample of refugees with a history of suicidality for this type of investigation. However, the manuscript presents several methodological and reporting issues that should be addressed to improve clarity and interpretability. In particular, the Methods section would benefit from a more detailed and coherent description. In addition, it remains unclear who exactly comprised the study sample, specifically whether the participants included only refugees with a history of suicidality or also the reported control group, which is no longer clearly referred to in the Results section. Last but not least, the English language and overall writing style should be revised throughout the manuscript, as several sentences are awkwardly phrased and difficult to follow.

Below is a list of comments and suggestions intended to help strengthen the manuscript.

Impact statement

“Sub-Saharan Africa reported the highest suicide mortality in 2021, and hence, significantly contributes to the deaths that make low- and middle-income countries (LMICs) the most affected, registering 73% of the global suicide burden”.

The authors should clarify what is meant by “73% of the global suicide burden.” As currently phrased, the statement could be misinterpreted as indicating that Sub-Saharan Africa accounts for 73% of all global suicides, which appears unlikely. This issue should also be corrected in the Introduction.

Introduction

The statement appears somewhat inconsistent. Claiming that the suicide rate “has not decreased significantly over the past 10 years” seems difficult to reconcile with the subsequent statement that it “decreased by 35% over 20 years,” unless the authors clarify that most of the reduction occurred earlier in the period and that progress has plateaued more recently.

“This pace will not be sufficient to achieve the target of 3.4.2, which calls for a 33% reduction in the suicide rate by 2030 compared with 2015 (WHO, 2026).”

The reference to “target 3.4.2” would benefit from greater specificity and contextualization, as readers may not be familiar with the SDG indicator numbering system. Consider explicitly referring to Sustainable Development Goal (SDG) Target 3.4 and its suicide mortality indicator (3.4.2), which aims to reduce suicide rates by one third by 2030 compared with 2015 levels.

“Previous evidence indicates that the age-standardized suicide rate has decreased from 8.9 per 100,000 people in 2021 to 9 per 100,000 people in 2019 (International Association for Suicide Prevention [IASP], 2022).”

The sentence is unclear and appears internally inconsistent, as it describes a “decrease” from 8.9 per 100,000 in 2021 to 9 per 100,000 in 2019, which reverses the chronological order and implies a numerical increase rather than a decrease. The authors should revise the sentence for clarity and ensure that the temporal sequence and direction of change are accurately reported.

Methods

The description “356 controls of suicide attempts” is unclear. I assume that the main inclusion criterion for the control group was being a refugee without a history of suicide attempts; if so, this should be stated explicitly.

The determination of the sample size is described repeatedly throughout the manuscript. Consider streamlining these sections to improve conciseness and optimize space. In addition, the original article by Krejcie and Morgan (1970) should be cited at the first mention of the method.

“For the qualitative study, purposive sampling was used to sample three participants per refugee. First, all settlements were represented at the point of data saturation. Data saturation was not reached for each case in the first round of interviews; therefore, another round was conducted. However, saturation was set when the first settlement (Rhino camp) conducted interviews (when a total of 15 had been conducted).”

The description of the qualitative sampling and data saturation procedures is difficult to follow and appears somewhat inconsistent. In particular, it is unclear what is meant by “three participants per refugee,” how saturation was operationalized across settlements, and how data saturation could be determined based on interviews conducted in the first settlement (Rhino camp) while all settlements were reportedly represented. The authors should clarify the sampling strategy and provide a more coherent description of the saturation process.

Was the Coping Strategies Inventory (CSI) translated and validated for the refugee population in which it was applied? If so, the authors should provide the relevant validation reference. If not, they should clarify how the linguistic and cultural adaptation of the instrument was performed.

Results

“Table 1 shows that three-quarters of the 54(75.0%) of those refugees were female, aged between 40 and 50 years, and 54(75.0%).”

This sentence is unclear and internally inconsistent (“54 [75.0%]” is repeated, and the structure makes the demographic characteristics difficult to interpret). Please reformulate the sentence for clarity.

The statement “a quarter (17%, n = 12) of refugees who had attempted suicide coped adaptively” would benefit from greater methodological clarification. The criteria used to classify participants as coping “adaptively” should be explicitly predefined and described in the Methods section.

Before conducting Principal Component Analysis (PCA), it is important to verify that the data are suitable for dimensionality reduction. Two commonly used preliminary tests are the Kaiser–Meyer–Olkin (KMO) measure and Bartlett’s test of sphericity. The KMO statistic assesses sampling adequacy and indicates whether the variables share enough common variance to justify PCA; values above 0.5 are generally considered acceptable, whereas values above 0.7 indicate good adequacy. Bartlett’s test evaluates whether the variables are sufficiently intercorrelated for factor extraction, with a statistically significant result (p < 0.05) supporting the use of PCA. Together, these tests help determine whether PCA can be meaningfully applied to the dataset. This information should be presented before reporting the results of the PCA, as it provides the rationale for the suitability of the data for dimensionality reduction. In addition, the authors should report the scree plot used to determine the number of components to retain.

It is unclear which variables were included in the cluster analysis. Were the 11 extracted principal components used as input variables? In addition, the authors should specify the clustering procedure applied and describe the method used to derive the clusters. Indeed, the authors should provide more detail regarding the specific k-means clustering procedure that was applied. As “k-means” encompasses several algorithmic variants and implementation strategies, it would be important to specify the distance metric used (typically Euclidean distance), the centroid initialization method (e.g., random initialization or k-means++), and the optimization routine adopted (e.g., Lloyd or Hartigan–Wong algorithm). In addition, the manuscript should clarify how the optimal number of clusters was determined (e.g., elbow method, silhouette analysis, gap statistic) and whether any procedures were used to assess cluster stability or robustness.

The Columbia Suicide Severity Scale (CSSS) is mentioned in Figure 1 but is not described in the Methods section. The authors should provide information on the instrument, including whether a validated version was available for the target population. If no validated version existed, the procedures used for linguistic and cultural adaptation of the original CSSS should be described.

“The findings in Table 3 show that in the largest cluster of refugees who had attempted suicide, the largest proportion coped by rarely accepting sympathy and understanding from 18 (37.5%), frequently trying to keep their feelings to themselves 24(50.0%), realizing that they brought the problem to themselves 18(37.5%), spending more time alone 30(62.5%), ...”.

The number and percentage should be reported together in parentheses using a consistent format, for example: (18 [37.5%]).

Discussion

“Most of the refugees (8 in every 10) attempted suicide by maladaptive coping with the aftermath of the mental health challenges, implying that they are at a very high risk of repeated suicide attempts and potentially suicide, given that they are not coping with the cause of the problem.”

This sentence would benefit from reformulation, as the causal and conceptual relationships are currently unclear. In particular, the expression “attempted suicide by maladaptive coping” is difficult to interpret, and the subsequent inference regarding a “very high risk” of repeated suicide attempts or suicide appears overly strong unless supported by longitudinal evidence. Consider using more precise and cautious language.

Additional comments

English style and grammar should be improved throughout the manuscript, as several sentences are awkwardly phrased. For example (Impact statement): “This is evidence of the need to implement more suicide prevention efforts in Africa, which can take several routes, perhaps the most important yet usually sidelined being the promotion of adaptive coping for all refugees with a history of suicidal ideation or attempts”.

# Reviewer report: Review: Coping mechanisms adopted following a suicide attempt among refugees in humanitarian settings within northern Uganda — R0/PR4

**DOI:** 10.1017/gmh.2026.10263.pr4

**Authors:** Krystyna Loney

This is an interesting study looking at the association between coping mechanisms and suicidal behaviour in refugees. I think this will be an important addition to the literature. However, there are weaknesses within the manuscript which need to be addressed. I have provided comments, which I hope will help the authors to strengthen the manuscript.

Abstract

The opening statement of the abstract comes across as judgemental. It also is not necessarily true. Some people will act impulsively on their thoughts before making a suicide attempt, without prior histories of suicidal thoughts or behaviours. Also the way your sentence is worded suggests that coping mechanisms are the only deciding factor between making and attempt and not making an attempt, which is not accurate. I suggest rewording this.

The statement within the results section within your abstract “…implying that they are at a very high risk of repeated suicide attempts and potentially suicide.” Your study does not appear to be measured over time and does not assess the outcome of repeat suicide attempt so you cannot jump to this conclusion. You are inferring too much from the results you found and must be much more cautious about making these bold statements.

Introduction

In general, please make sure that you reference correctly and consistently throughout your introduction. There are a number of comments which should be referenced and are not.

I would advise providing further rationale for why deficits in coping are risk factors for suicidal thoughts or behaviors. You could use theoretical models to show this. E.g IMV Model of Suicidal Behaviour.

You mention that coping mechanisms are “significant for suicide prevention” online 47. Please expand on how they are significant for suicide prevention. You need to provide more information to give the reader a better vision of why you are conducting this study, what it will add to the literature, why it is important etc.

Page 5, Line 51-54 “However, despite the numerous assessments of suicide attempts and ideation done so far among those refugees” I don’t know what you mean by this statement? Please reword.

Page 5, Line 54-56 “the issue of how refugees who have ideation and survive suicidal attempt cope has not been studied much”. You need to give more information on what we already know and what the gaps are. If coping in refugees with a history of suicide attempt has not been studied much, then tell me what exactly has been done, what hasn’t? Provide the rationale for why your study fills a gap and be clear about why.

Overall, the introduction required further refined rationale for what we know about coping in refugees with suicide attempt history already, as well as why this specific study is important to conduct.

Methods

The methods section is unclear. Please split your methods into quant and qual, including information on recruitment, participant eligibility, and procedures, measures used etc. There are repetitions of some parts of information, while other methods are unclear and confusing. If I was to replicate this study I would not have adequate information to do so.

I am unclear how many participants are and what their histories are. How many were included in the quan analysis? How many in the qual? Also did those who took part in the study have a history of suicidal thoughts, attempt or both? You must be clear about the outcome. Also how did you assess history of suicidal thoughts or behaviours? Was it a clinical interview, did you use a validated measure?

Page 6 Line 47-49 “Given that coping mechanisms were practically lived experience of those who had ever ideated or attempt suicide” I’m not sure what this sentence means. Everyone uses coping mechanisms to cope with stressors in their lives? Please rephrase.

Page 6, Line 55 “…all of which registered more than 70% of suicide attempt cases reported…” I am unclear about this statement, does this mean only 70% of your sample had made a suicide attempt?

Please give information on how data was collected. After the audio recording were taken during interview, were these transcribed verbatim? Did the researchers do this or did an external company?

Page 8, line 16 “…data saturation was not reached for each case in the first round of interviews; therefore another round was conducted”. Do you mean recruitment was re-opened and more interviews were conducted after the first round of recruitment?

Results

It is unclear to me why a principal component analysis was conducted. The reason for this choice of analysis needs to be explained.

Page 9, line 49 “… and 54 (75%)” this is a typo, you said this earlier in the sentence.

Page 10, line 41 “…The largest of these clusters was cluster 1…” Was is this cluster? Results need to be explained more.

Page 11, line 32 “Similar to how most refugees try to cope with suicidal ideation…” How do you know that most refugees cope in this way? You can’t make this statement without having evidence.

Discussion

Start the discussion by summarising what your study found to be clear to the reader.

The discussion section of this manuscript needs some attention. There are numerous topics which are raised in the discussion which were not explained in the introduction (e.g. transaction model of stress and coping, you mention emotion-focused, problem-focused etc which haven’t been put into context in your intro). Your discussion needs to detail how your results sit in the wider context of the literature, which you should already have laid out in your introduction.

Your discussion makes some bold statements which cannot be inferred from your results. Be very clear about what exactly your results contribute, instead of jumping to conclusions.

Page 13, line 47-56, a number of statements made in this section are not referenced correctly. If you make a statement you must be able to back it up as evidence.

Not sure how migraine headaches relate to this study? I would suggest removing this section.

Your discussion needs to mention both quant and qual findings. It is difficult to read which parts refer to discussion of each of your findings.

I would suggest adding a section on implications of this research and what it means for moving forward.

Supplementary materials

Authors are referring to ideation and suicide attempt interchangeably. Please state which outcome you are referring to for each table.

Similar to my comment about the cluster analysis results, the graph in the supplementary materials needs explained. What are cluster 1 and 2

General comments

Please be clear about your outcome throughout

Please be clear about how you are defining coping. Sometimes specific strategies are mentioned, sometimes ‘adaptive’ ‘maladaptive’ mentioned. There is no consistent terminology or description of coping.

Be careful with your phrasing throughout. At times it comes across as stigmatising.

# Editor report: Recommendation: Coping mechanisms adopted following a suicide attempt among refugees in humanitarian settings within northern Uganda — R0/PR5

**DOI:** 10.1017/gmh.2026.10263.pr5

**Affiliations:** Duke University School of Nursing, United States

Thank you for submitting this interesting and timely manuscript. The reviewers agreed that this manuscript addresses an important and clinically relevant topic, and the research is relevant to the journal. However, they also agreed that several revisions are necessary to prepare the work for publication, particularly in the reporting of the methods and study findings. This included a clearer description of the study sample and control group, as well as a close editorial review to ensure the writing is clear and accurate, and that the references are improved throughout the manuscript. Finally, Reviewer 2 felt that the discussion made some overstated claims that were not supported by the study findings, and I agree with their assessment.

# Editor report: Decision: Coping mechanisms adopted following a suicide attempt among refugees in humanitarian settings within northern Uganda — R0/PR6

**DOI:** 10.1017/gmh.2026.10263.pr6

**Affiliations:** London School of Hygiene & Tropical Medicine, United Kingdom of Great Britain and Northern Ireland

No accompanying comment.

# Reviewer report: Review: Coping mechanisms adopted following a suicide attempt among refugees in humanitarian settings within northern Uganda — R1/PR8

**DOI:** 10.1017/gmh.2026.10263.pr8

The authors have addressed most of the reviewers’ comments, and the manuscript has improved accordingly.

# Reviewer report: Review: Coping mechanisms adopted following a suicide attempt among refugees in humanitarian settings within northern Uganda — R1/PR9

**DOI:** 10.1017/gmh.2026.10263.pr9

**Authors:** Krystyna Loney

The revisions made by the authors have improved the m/s. However, I have further comments.

As per my previous comment, the opening statement does not read well. Adding in the word ‘usually’ does not change the meaning of the sentence and I would still recommend rewording as the language appears stigmatising.

There are still uncertainties around the methods. The methodology section should at minimum be broken down into participants, procedure, measures, and statistical analysis section. As per my previous comment, despite it being mentioned how many participants you aimed to recruit there is no clear section detailing this information.

Furthermore, the measures used to assess suicide attempt is not clear. Authors mention that C-SSS was used, but this scale measures both ideation and attempt, so did this m/s only use the items related to attempt? If the entire scale was used, then both ideation and behaviour were measured, and the outcome of the study must be labelled as such.

Also, all measures should be in one section together. At present suicide attempt measure is mentioned early in the methods, while coping later mentioned. These should be placed together.

As per my previous comment to rephrase this sentence “Given that coping mechanisms were practically lived experience of those who had ever ideated or attempt suicide” – your rephrased sentence of ‘given that coping mechanisms are practically lived experiences of persons with a history of attempted suicide’ is also not at all clear. This is one example, but my opinion is that the general writing style of the entire manuscript should be refined to improve the clarity and readability.

As per my previous comment, I am unclear how migraine headaches are relevant to this specific study. The mention of it in this m/s confuses the reader and detracts from the important findings and implications that have been identified.

As per my previous comment, discussion of quant and qual results is unclear. If there was congruence between the mixed-method findings, then that is a point worth mentioning as part of your discussion.

# Editor report: Recommendation: Coping mechanisms adopted following a suicide attempt among refugees in humanitarian settings within northern Uganda — R1/PR10

**DOI:** 10.1017/gmh.2026.10263.pr10

**Affiliations:** Duke University School of Nursing, United States

We thank the authors for their efforts to revise this manuscript, which have resolved the majority of the issues raised during the first round of peer review. However, one of the reviewers has suggested some additional revisions, and I agree with that assessment.

Specifically, the authors responded to several of the comments in their response letter, but did not make corresponding revisions in the manuscript to add or clarify those comments. See specific examples, with particular attention to the methods section and descriptions of measures, as well as improved comparison/contrast/integration of the quantitative and qualitative findings in the discussion.

# Editor report: Decision: Coping mechanisms adopted following a suicide attempt among refugees in humanitarian settings within northern Uganda — R1/PR11

**DOI:** 10.1017/gmh.2026.10263.pr11

**Affiliations:** London School of Hygiene & Tropical Medicine, United Kingdom of Great Britain and Northern Ireland

No accompanying comment.

# Editor report: Recommendation: Coping mechanisms adopted following a suicide attempt among refugees in humanitarian settings within northern Uganda — R2/PR13

**DOI:** 10.1017/gmh.2026.10263.pr13

**Affiliations:** Duke University School of Nursing, United States

The reviewer comments have now been adequately addressed and I believe the manuscript is suitable for publication. However, I did notice some minor typographical errors. 

On page 9, the acronym “C-SRS” is used and should be “C-SSRS”. 

In the newly added text on page 15, the parentheses should be added to read: “Isolation (frequently trying to keep their feelings to themselves, realizing that they brought the problem to themselves, spending more time alone), Actively preventing attempt causes (standing on their ground and fighting for what they wanted), and Self-blame...”

# Editor report: Decision: Coping mechanisms adopted following a suicide attempt among refugees in humanitarian settings within northern Uganda — R2/PR14

**DOI:** 10.1017/gmh.2026.10263.pr14

**Affiliations:** London School of Hygiene & Tropical Medicine, United Kingdom of Great Britain and Northern Ireland

No accompanying comment.
